# Assessing the mental effects of COVID-19-related work on depression among community health workers in Vietnam

**DOI:** 10.1186/s12960-022-00760-x

**Published:** 2022-08-19

**Authors:** Ngoc-Anh Hoang, Ngoc Van Hoang, Ha-Linh Quach, Khanh Cong Nguyen, Luong Huy Duong, Thai Quang Pham, Florian Vogt

**Affiliations:** 1 National Centre for Epidemiology and Population Health, Research School of Population Health, College of Health and Medicine, Australian National University, Canberra, ACT Australia; 2 National Institute of Hygiene and Epidemiology, Hanoi, Vietnam; 3 The General Department of Preventive Medicine, Ministry of Health, Hanoi, Vietnam; 4 Medical Services Administration, Ministry of Health, Hanoi, Vietnam; 5 The Kirby Institute, University of New South Wales, Sydney, NSW Australia

**Keywords:** COVID-19, Depression, Community health workers, Vietnam

## Abstract

**Background:**

Community health workers (CHWs) involved in the COVID-19 response might be at increased risk of developing depression, though evidence is scarce. We investigated effects of COVID-19-related work on changes in depression levels among CHWs in Vietnam and identified sub-groups among CHWs who are at particular risk of developing severe depression.

**Methods:**

We conducted a cross-sectional online survey among 979 CHWs who were involved in the COVID-19 response in Vietnam, in particular during the 2021 Tet holiday outbreak between January and March 2021. Respondents were asked to report depression symptoms at two-time points, before the start of the COVID-19 pandemic (average June to December 2019) and during the 2021 Tet holiday outbreak using the PHQ-9 mental health questionnaire. We estimated depression levels at both time points and developed univariate and multivariable logistic regression models to estimate odds ratios and 95% confidence intervals (95% CIs) to explore the association between deterioration to high depression levels and selected risk factors.

**Results:**

Median depression levels among CHWs in Vietnam doubled from 3 (IQR = 2–7) before COVID-19 to 6 (IQR = 3–9) on the PHQ-9 scale during the Tet holiday outbreak. The proportion with normal/minimal levels decreased from 77.1% (95% CI = 74.4–79.7) to 50.9% (95% CI = 47.7–54) (*p*-value < 0.001), while the proportion of CHWs with moderate, moderately severe, and severe depression levels increased 4.3, 4.5, and five-fold, respectively. Less sleep and poor sleep quality, working in unfavorable work environments, and being involved in contact tracing and the organization of quarantine for suspected cases were associated with an increased risk of deterioration to high depression levels.

**Conclusions:**

We found a substantial increase in overall depression levels among CHWs in Vietnam due to their COVID-19 related work and a particularly worrisome rise in CHWs suffering from severe depression. CHWs are an indispensable yet often overlooked cadre of work in many low- and middle-income countries and shoulder a heavy psychological burden during the COVID-19 pandemic. Targeted psychological support for CHWs is needed to improve their mental health and to ensure the sustainability of community-based health interventions during COVID-19 and future epidemics.

**Supplementary Information:**

The online version contains supplementary material available at 10.1186/s12960-022-00760-x.

## Background

Community health workers (CHWs) constitute an important cadre of the health care workforce in the response to COVID-19 in low- and middle-income countries [1]. In Vietnam, CHWs provide culturally appropriate health education and information, assist people at community level to get the care they need, give counselling and guidance on health behaviors, and provide some direct health services, such as first aid and blood pressure screening [2]. The COVID-19 pandemic has placed CHWs in an unprecedented situation; their tasks expanded substantially into areas outside their usual, pre-pandemic activities. The most common activities include pandemic front line work such as raise community awareness about COVID-19, conduct screening and testing in the community, organize quarantine and isolation, and trace close contacts of COVID-19 cases.

There is evidence suggesting that clinical cadre of the health care workforce like nurses and doctors are at considerable risk of developing mental illness due to their work with COVID-19 patients [3, 4], with depression being among the most frequent disorders. For example, in studies from China by Lai et al. and by Chen et al., nearly half of health care workers who were exposed to COVID-19 patients reported symptoms of depression [5, 6]. Similarly, in a pooled study by Olaya et al., depression prevalence in front line health care workers during COVID-19 from 57 studies was 43% [7]. Another study by Kang et al. among medical and nursing staff in Wuhan, China, identified 37% of clinical staff as suffering from depression [8]. In Vietnam, reported rates of clinical staff who were involved in the COVID-19 response and suffered from some degree of depression range between 17 and 35% [9–11]. Working in high-risk areas, in close contact with SARS-CoV-2 patients, long working hours, high workload, shortage of sleep, and poor working conditions have been suggested as risk factors for developing COVID-19-related depression among clinical staff [12, 13]. However, all existing evidence about the link between COVID-19-related work and mental health is limited to staff performing patient-centered clinical duties in health facilities. Evidence from non-clinical health care workers outside health facilities such as CHWs remains limited [14]. To our knowledge, the mental health effects of COVID-19-related work among CHWs has not been quantified to date.

Aiming to address this gap, we assessed the effects of COVID-19-related work on depression among CHWs in Vietnam by comparing baseline depression levels before the COVID-19 pandemic and during the 2021 Tet holiday outbreak, a major nationwide COVID-19 outbreak during January and March 2021. We also aimed to determine associated factors in order to identify sub-groups among CHWs that might be at particular high risk of suffering a deterioration to high depression levels.

## Methods

### Study design

We conducted a cross-sectional online survey among CHWs in Vietnam who were involved in the COVID-19 response during the 2021 Tet holiday outbreak. The survey comprised questions about two time points: the pre-pandemic baseline period (6-month average June to December 2020) and the period of the 2021 Tet holiday outbreak (average January to March 2021).

### Study setting

**Fig. 1:**
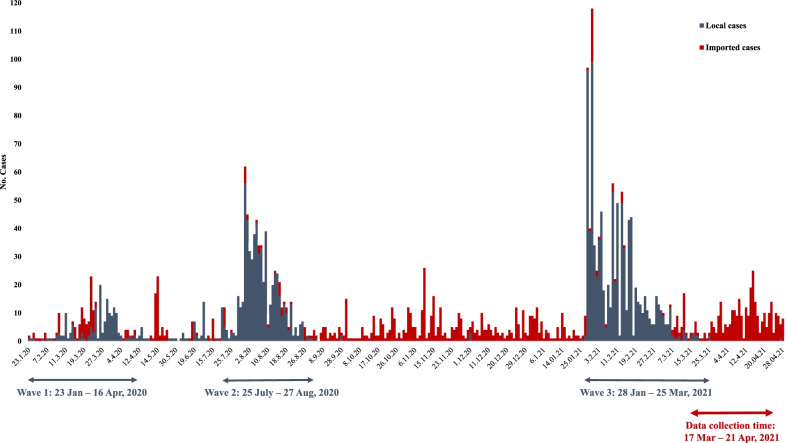
Daily COVID-19 cases in Vietnam, January 2020 to April 2021

**Fig. 2:**
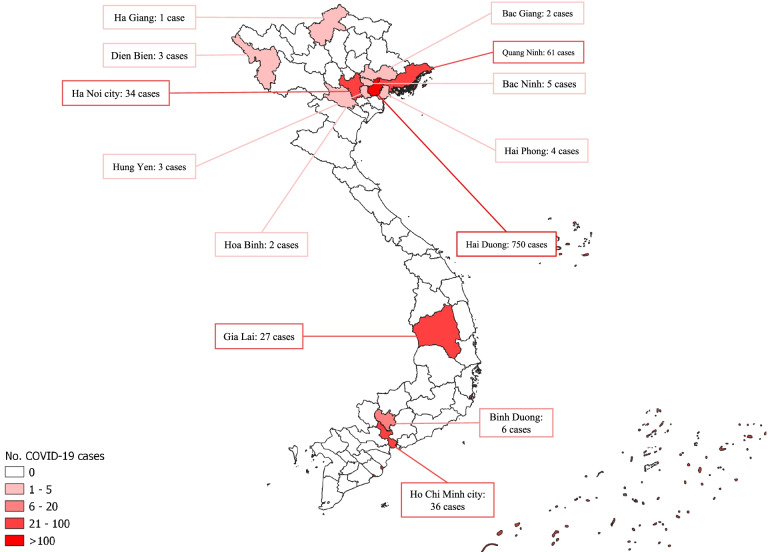
COVID-19 cases in Vietnam by province during the Tet holiday outbreak (28 January–25 March 2021)

Until March 2021, Vietnam experienced three waves of COVID-19 (Fig. 1). The first wave, which was dominated by imported cases among returning travelers, lasted until mid-April 2020 with a total of 106 cases. The second wave was characterized by local transmission in the community and hospitals during mid-2020 and resulted in a total of 554 cases. On 28 January 2021, the third, and until, the largest wave (also referred to as the “2021 Tet holiday outbreak”) started with the detection of a COVID-19 cluster among employees at an industrial complex in Hai Duong Province, Northern Vietnam. The outbreak spread quickly across 12 other provinces. CHWs got activated in virtually all provinces across Vietnam as part of massive outbreak response activities, which was eventually achieved on 25 March 2021 after resulting in 934 cases nationwide (Fig. 2). We conducted our survey shortly after the peak of the Tet holiday outbreak (Fig. 1).

Our study population were CHWs in Vietnam aged 18 years or older who were involved in the 2021 Tet holiday outbreak through at least one of the following activities: tracing of close contacts of confirmed or suspected COVID-19 cases; organizing quarantine for suspected COVID-19 cases and their contacts; screening for people with COVID-19 symptoms in the community; participating in any other activities that involved direct exposure to potential SARS-CoV-2 cases.

### Data collection

Shortly after the beginning of the COVID-19 pandemic in Vietnam, the Ministry of Health created a group on Zalo, a popular social media application for smartphones in Vietnam [15], to link CHWs across all 64 provinces and cities together. The aim of this group is to enable quick and informal peer-to-peer communication about COVID-19-related work, e.g., be up to date about contact tracing across provinces, and sharing of individual stories, knowledge, and experiences. About 200 CHWs had joined this group at the time of our study. We obtained permission from the national-level administrators of this group to invite its members to complete a short online survey. Completing the survey was entirely voluntary, confidential, and anonymous. We created the survey using the KoBoToolbox [16] and piloted the questionnaire prior to dissemination to ensure appropriateness, clarity, user-friendliness, and a realistic completion time of approximately 15 min. The survey was opened on 17 March 2021 by posting an invitation on Zalo with a participant information sheet and a link to the online questionnaire (Additional file 1). CHWs were encouraged to disseminate the survey link to other CHWs outside the group. We posted two reminders 2 and 4 weeks after the first invitation. The link was deactivated on 21 April 2021.

### Variables

#### Outcome of interest

We used the Vietnamese version of the Patient Health Questionnaire (PHQ-9) to quantify depression levels among respondents (Additional file 1). The PHQ-9 has been in widespread use around the world since its development in 1999 [17] and its Vietnamese version has been used in several previous studies [18–21]. The PHQ-9 contains nine items about depression-related symptoms. Each item has four response categories regarding their frequencies (> 4 days/week, 3–4 days/week, 1–2 days/week, not at all) resulting in a total range score from 0 to 27. The recommended cut-off points for depression thresholds are as follows: normal/minimal (0–4), mild (5–9), moderate (10–14), moderately severe (15–19), and severe (20–27) [22]. We asked about self-reported depression levels at two time points: 6 months before the pandemic and during the 2021 Tet holiday outbreak. For the regression models, the outcome variable “deterioration to high depression levels” was created. People who reported normal/minimal, mild, or moderate depression levels before the pandemic and an increase to moderately severe or severe levels during the 2021 Tet holiday outbreak; and those with moderately severe depression levels before the start of the pandemic and an increase to severe levels during the 2021 Tet holiday outbreak were categorized as having deteriorated to high depression levels.

#### Co-variates

The variables collected on socio-demographic characteristics were age, sex, marital status, living with children under the age of five or with elderly persons, socio-economic status (SES), and years of work experience. We also asked about pre-existing physiological or mental health conditions, and other acute medical issue, as well as the number of hours of sleep per day and quality of sleep. Questions about work conditions referred to changes in feeling overloaded before the pandemic and during the 2021 Tet holiday outbreak; estimated working hours per day; working overtime, working on weekends; the number of paid workplaces for respondents; and five questions on their overall perceived working environment. Work-related intensity of exposure to SARS-CoV-2 was assessed through four questions on the estimated weekly frequencies of four activities: (1) the participation in contact tracing/case finding; (2) the organization of quarantine for suspected cases and close contacts; (3) the screening for COVID-19 symptoms in the community by taking swab samples or measuring temperature; and (4) any other activities that required direct exposure to potential SARS-CoV-2 cases. Using the national COVID-19 incidence database, we also calculated the number of confirmed COVID-19 cases per province during the 2021 Tet holiday outbreak to categorize CHWs into three groups: CHWs working in provinces with zero confirmed case; with low case numbers (< 27 cases); or high case numbers (≥ 27 cases). See Additional file 1: Supplement 1.1 for the full questionnaire and Additional file 2 for details on variable management.

### Sample size

This was an exploratory study without hypothesis testing, hence no formal sample size was calculated. In total, 979 participants submitted sufficient data to be included in the analysis, which corresponds to 6% of the approximated total 15,600 CHWs in Vietnam [23].

### Data analysis

Data were extracted from the KoBo Toolbox software into Excel for management and imported to STATA 16.0 for cleaning and analysis. First, we tabulated the socio-demographic characteristics, pre-existing health conditions, exposure intensity to SARS-CoV-2, sleep conditions, and work conditions by outcome status (deterioration to high depression levels), and used Wilcoxon rank-sum tests, Pearson’s Chi-squared tests, and Fisher’s exact tests to detect statistically significant differences. We used box plots to compare baseline depression levels before the start of the COVID-19 pandemic with levels during the 2021 Tet holiday outbreak and used Spearman’s correlation coefficient to measure the association between depression levels at these two time points. We calculated the prevalence and 95% confidence intervals (95% CIs) of depression levels across the five severity categories pre-COVID-19 (baseline) and during the 2021 Tet holiday outbreak. We also created a heat map to illustrate distributions across the five depression levels at the two time points. We then used univariate and multivariable logistic regression to estimate odds ratios (ORs) and 95% CIs of the associations between co-variants (socio-demographic characteristics, health condition, intensity to exposure to SARS-CoV-2 sources, sleep condition, and work condition) and deterioration to high depression levels. We developed four multivariable logistic regression models to explore differences in the association of covariates and the outcome when adjusting for different sets of risk factors. The Base Model (Model 0) included socio-demographic characteristics and pre-existing health conditions as independent variables. In Model 1, we added work-related exposure intensity to SARS-CoV-2; the addition of sleep/work conditions with and without exposure intensity resulted in Models 2 and Model 3, respectively. The effect of each variable in the models was calculated using likelihood-ratio tests. We then used Akaike’s Information Criteria (AIC) and Schwarz’s Bayesian Information Criteria (BIC) to compare the fit among these four models [24].

## Results

**Fig. 3:**
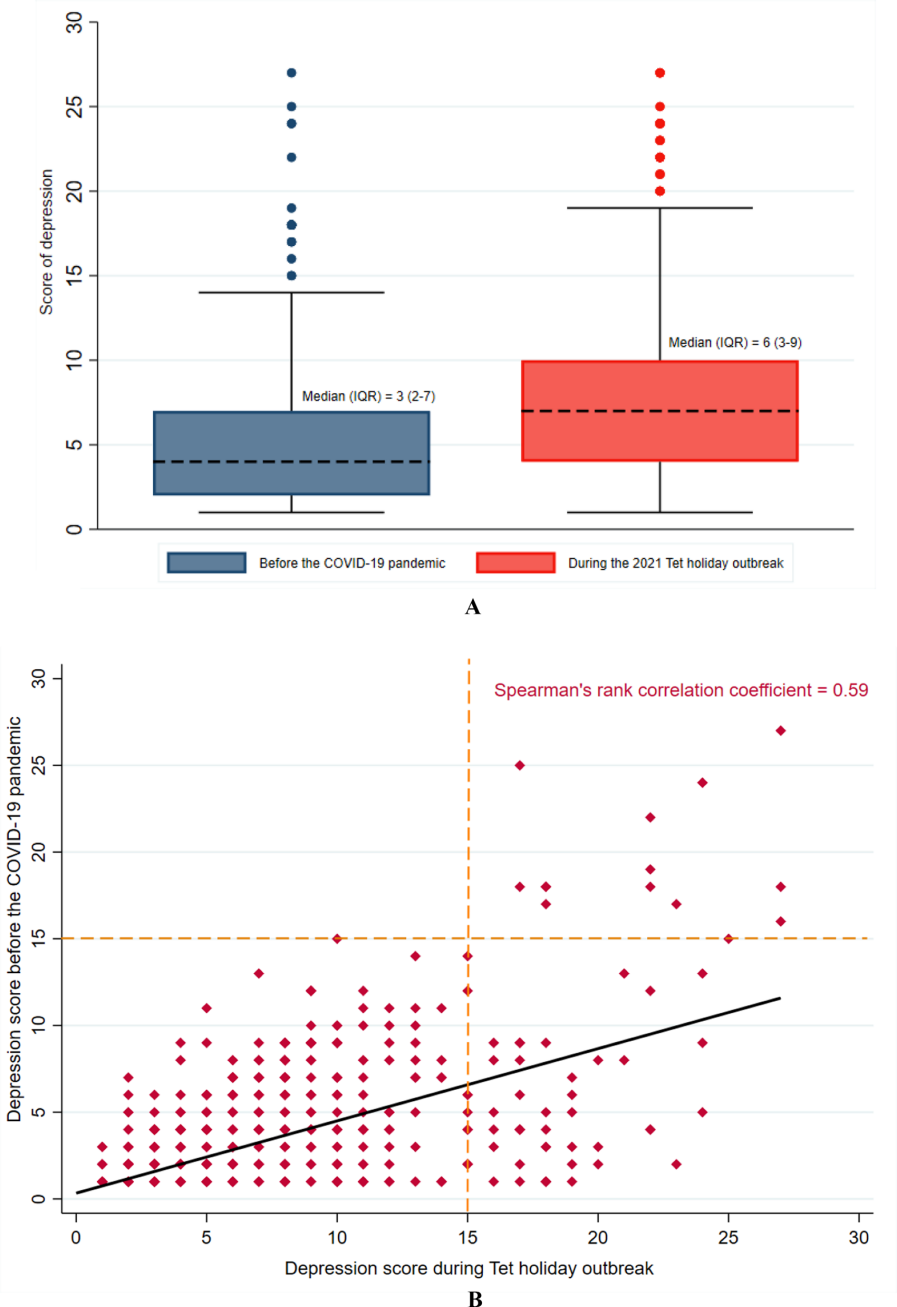
**A** Depression scores before the COVID-19 pandemic and during the 2021 Tet holiday outbreak among study participants. **B** Correlation of depression scores before the COVID-19 pandemic and during the Tet holiday outbreak among study participants. The solid black line represents the least squares regression line

A total of 979 participants were included in the final analysis. Of these, 71 participants (7.3%) showed deterioration to high depression levels. The median PHQ-9 score increased from 3 (IQR 2–7) from before the pandemic to 6 (IQR 3–9) during the 2021 Tet holiday outbreak (Fig. 3A). We detected a moderate positive correlation between the depression scores at both time points, with a Spearman’s rank correlation coefficient of 0.59 (Fig. 3B).

**Fig. 4:**
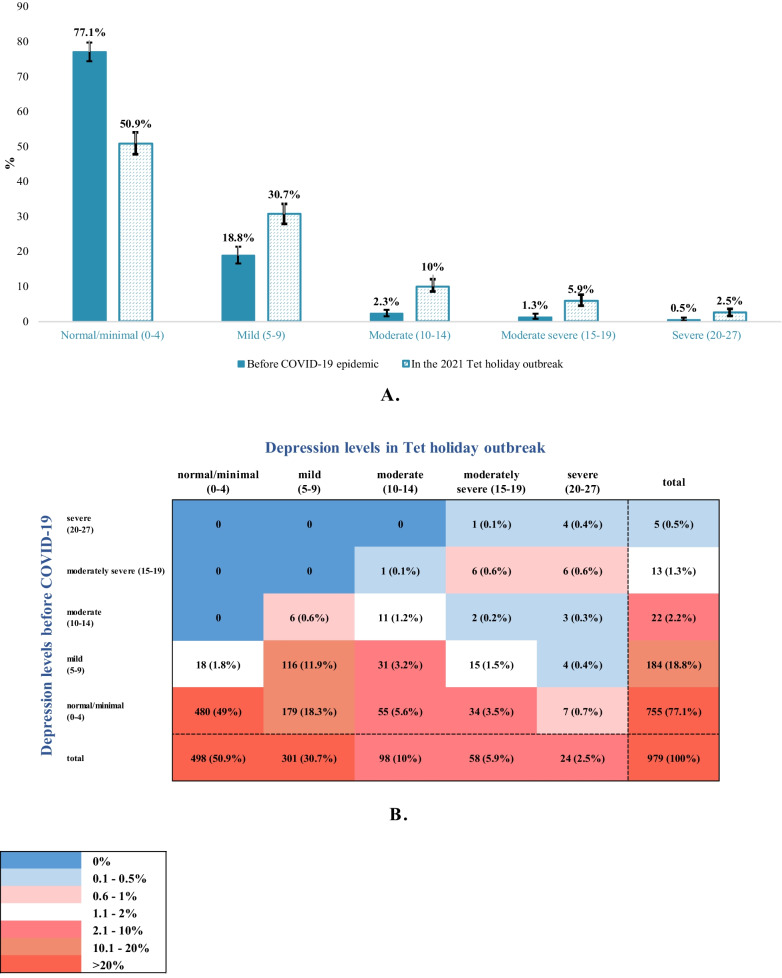
**A** Categorized depression levels before the COVID-19 pandemic and during the 2021 Tet holiday outbreak among study participants. The vertical error bar indicates the 95% confidence intervals. **B** Heat map of categorized depression levels before the COVID-19 pandemic and during the 2021 Tet holiday outbreak among study participants

The prevalence of depression symptoms was higher in every depression severity category during the Tet holiday outbreak compared to before the COVID-19 pandemic. While the majority (77.1%, 95% CI = 74.4–79.7) of CHWs showed no relevant depression levels pre-COVID, only about half (50.9%, 95% CI = 47.7–54) were free of depression during the Tet holiday outbreak (*p*-value < 0.001). Mild depression symptoms were present in 18.8% (95% CI = 16.5–21.4) of respondents before COVID-19 compared with 30.7% (95% CI = 27.9–33.7) during the Tet holiday outbreak (*p*-value < 0.001); moderate depression symptoms prevalence was 2.3% (95% CI = 1.5–3.4) compared with 10% (95% CI = 8.3–12.1) (*p*-value < 0.001); moderately severe depression symptom prevalence was 1.3% (95% CI = 0.8–2.3) compared with 5.9% (95% CI = 4.5–7.6%) (*p*-value < 0.001); and severe depression prevalence was 0.5% (95% CI = 0.2–1.2) compared with 2.5% (95% CI = 1.6–3.6) (*p*-value < 0.001) (Fig. 4A and Table 1). This corresponds to a 1.6-fold increase for mild depression levels, a 4.3-fold increase for moderate depression levels, a 4.5-fold increase for moderately severe depression levels, and a fivefold increase for severe depression levels during the Tet outbreak compared with before COVID-19 (Table 1). The prevalence of CHWs with normal/minimal depression levels before COVID-19 and changed to moderate, moderately severe, severe symptoms during the Tet holiday outbreak was 5.6%, 3.5%, and 0.7%, respectively. This figure among CHWs with mild depression levels pre-COVID was 3.2%, 1.5%, and 0.4% (Fig. 4B).

**Table 1:** Comparison of depression levels before the COVID-19 pandemic and during the 2021 Tet holiday outbreak among study participants

Depression levels	% (95% CI)				% difference	
	Before COVID-19 pandemic	During the 2021 Tet holiday outbreak	*p*-value*	Absolute		Relative
Normal/minimal (0–4)	77.1 (74.4–79.7)	50.9 (47.7–54)	**< 0.001**	− 26.2		0.7
Mild (5–9)	18.8 (16.5–21.4)	30.7 (27.9–33.7)	**< 0.001**	11.9		1.6
Moderate (10–14)	2.3 (1.5–3.4)	10 (8.3–12.1)	**< 0.001**	7.7		4.3
Moderately severe (15–19)	1.3 (0.8–2.3)	5.9 (4.5–7.6)	**< 0.001**	4.6		4.5
Severe (20–27)	0.5 (0.2–1.2)	2.5 (1.6–3.6)	**< 0.001**	2		5

*p-value from Fisher’s exact test

The comparison of covariates by the main outcome (deterioration to high depression levels) is shown in Tables 2, 3, 4. The median age of participants was 37 (interquartile ranges [IQR]: 32–45). More than half of CHWs were female (52%) and were living with children under the age of five or with elderly (65.9%). Most of the participating CHWs were married (89.4%), had no pre-existing long-term physiological health problems (84.9%), had no pre-existing mental health disorders (96.6%), and had no acute/sudden-onset medical problems (96.6%). CHWs who deteriorated to high depression levels were more likely to be younger [median (IQR): 35 (31–42) vs. 38 (32–45), *p*-value = 0.026], had fewer years of job experience (less than five years: 15.2% vs. 26.8%), had a lower SES level (32.5% vs. 49.3%, *p*-value = 0.009), and had more pre-existing diagnosed mental health disorders (3.1% vs. 7%, *p*-value = 0.08) compared to those who did not deteriorate to high depression levels.

**Table 2:** Socio-demographic characteristics and health conditions of study participants by deterioration to high depression levels

Variables	Total	Deterioration to high depression levels		*p*-value^*^
		No	Yes	
	*n* (%)	*n* (%)	*n* (%)	
Total	979 (100)	908 (92.7)	71 (7.3)	
Sociodemographic				
Age [median (IQR)]	37 (32–45)	38 (32–45)	35 (31–42)	**0.026**
Age groups				
< 30 y	116 (11.8)	103 (11.3)	13 (18.3)	0.300
30–34 y	242 (24.7)	221 (24.3)	21 (29.6)	
35–39 y	228 (23.3)	212 (23.3)	16 (22.5)	
40–44 y	130 (13.3)	123 (13.5)	7 (9.9)	
45–49 y	124 (12.7)	116 (12.8)	8 (11.3)	
50 +	139 (14.2)	133 (14.6)	6 (8.5)	
Sex				
Male	470 (48.0)	435 (47.9)	35 (49.3)	0.820
Female	509 (52.0)	473 (52.1)	36 (50.7)	
Marital status				
Single	84 (8.6)	76 (8.4)	8 (11.3)	0.470
Married	875 (89.4)	814 (89.6)	61 (85.9)	
Divorced/widow	20 (2.0)	18 (2.0)	2 (2.8)	
Living with children or elderly				
No	334 (34.1)	315 (34.7)	19 (26.8)	0.170
Yes	645 (65.9)	593 (65.3)	52 (73.2)	
Years of job experiences				
< 5 y	157 (16.0)	138 (15.2)	19 (26.8)	0.100
5–9 y	180 (18.4)	164 (18.1)	16 (22.5)	
10–14 y	294 (30.0)	278 (30.6)	16 (22.5)	
15–19 y	122 (12.5)	116 (12.8)	6 (8.5)	
20–24 y	99 (10.1)	92 (10.1)	7 (9.9)	
25 +	127 (13.0)	120 (13.2)	7 (9.9)	
Social economic status				
Upper	55 (5.6)	50 (5.5)	5 (7.0)	**0.009**
Middle	594 (60.7)	563 (62.0)	31 (43.7)	
Lower	330 (33.7)	295 (32.5)	35 (49.3)	
Health condition				
Pre-existing long-term physiological health problems				
No	831 (84.9)	772 (85.0)	59 (83.1)	0.660
Yes	148 (15.1)	136 (15.0)	12 (16.9)	
Pre-existing diagnosed mental health disorders				
No	945 (96.6)	880 (96.9)	65 (92.9)	0.080
Yes	33 (3.3)	28 (3.1)	5 (7.0)	
Missing value	1 (0.1)	0 (0)	1( 0.1)	
Acute/sudden-onset medical problems				
No	946 (96.6)	879 (96.8)	67 (94.4)	0.270
Yes	33 (3.4)	29 (3.2)	4 (5.6)	

*p-value from Wilcoxon rank-sum, Pearson’s Chi-squared and Fisher’s exact test

**Table 3:** Intensity of exposure to SARS-CoV-2 by deterioration to high depression levels among study participants during the 2021 Tet holiday outbreak

Variables	Total	Deterioration to high depression levels		*p*-value*
		No	Yes	
	*n* (%)	*n* (%)	*n* (%)	
Total	979 (100)	908 (92.7)	71 (7.3)	
Frequency of contact tracing per week				
Did not perform	292 (29.8)	277 (30.5)	15 (21.1)	0.180
Less than one day	63 (6.4)	60 (6.6)	3 (4.2)	
1–2 days	75 (7.7)	72 (7.9)	3 (4.2)	
3 days +	68 (6.9)	62 (6.8)	6 (8.5)	
Daily	481 (49.1)	437 (48.1)	44 (62.0)	
Frequency of organizing isolation per week				
Did not perform	402 (41.1)	381 (42.0)	21 (29.6)	0.290
Less than one day	68 (6.9)	62 (6.8)	6 (8.5)	
1–2 days	66 (6.7)	61 (6.7)	5 (7.0)	
3 days +	80 (8.2)	74 (8.1)	6 (8.5)	
Daily	363 (37.1)	330 (36.3)	33 (46.5)	
Frequency of screening for COVID-19 per week				
Did not perform	255 (26.0)	240 (26.4)	15 (21.1)	0.160
Less than 1 day	53 (5.4)	51 (5.6)	2 (2.8)	
1–2 days	78 (8.0)	75 (8.3)	3 (4.2)	
3 days +	67 (6.8)	58 (6.4)	9 (12.7)	
Daily	526 (53.7)	484 (53.3)	42 (59.2)	
Frequency of doing other activities had to expose to SARS-CoV-2 sources per week				
Did not perform	475 (48.5)	446 (49.1)	29 (40.8)	0.480
Less than 1 day	52 (5.3)	46 (5.1)	6 (8.5)	
1–2 days	73 (7.5)	67 (7.4)	6 (8.5)	
3 days +	66 (6.7)	60 (6.6)	6 (8.5)	
Daily	313 (32.0)	289 (31.8)	24 (33.8)	
Number of confirmed cases per province^†^				
None	743 (75.9)	696 (76.7)	47 (66.2)	0.130
< 27 cases	139 (14.2)	124 (13.7)	15 (21.1)	
≥ 27 cases	97 (9.9)	88 (9.7)	9 (12.7)	

*p-value from Fisher’s exact test

**Table 4:** Sleep and work conditions of study participants during the 2021 Tet outbreak by deterioration to high depression levels

Variables	Total	Deterioration to high depression levels		*p*-value*
		No	Yes	
	*n* (%)	*n* (%)	*n* (%)	
Total	979 (100)	908 (92.7)	71 (7.3)	
Sleep condition				
Hours of sleep per day				
7 + hours	474 (48.4)	465 (51.2)	9 (12.7)	**< 0.001**
Less than 7 h	505 (51.6)	443 (48.8)	62 (87.3)	
Quality of sleep				
Good	569 (58.1)	559 (61.6)	10 (14.1)	**< 0.001**
Normal	291 (29.7)	257 (28.3)	34 (47.9)	
Not good	119 (12.2)	92 (10.1)	27 (38.0)	
Work condition				
Working hours				
< 8 h	240 (24.5)	234 (25.8)	6 (8.5)	**< 0.001**
8–9 h	335 (34.2)	321 (35.4)	14 (19.7)	
9 + hours	404 (41.3)	353 (38.9)	51 (71.8)	
Work overtime in workdays				
No	121 (12.4)	117 (12.9)	4 (5.6)	0.090
Yes	858 (87.6)	791 (87.1)	67 (94.4)	
Work on weekends				
No	110 (11.2)	109 (12.0)	1 (1.4)	**0.003**
Yes	869 (88.8)	799 (88.0)	70 (98.6)	
Work overtime in workdays and work on weekends				
No	147 (15.0)	143 (15.7)	4 (5.6)	**0.023**
Yes	832 (85.0)	765 (84.3)	67 (94.4)	
Working in two places +				
No	837 (85.5)	784 (86.3)	53 (74.6)	**0.007**
Yes	142 (14.5)	124 (13.7)	18 (25.4)	
Job intensity				
Not intense	270 (27.6)	267 (29.4)	3 (4.2)	**< 0.001**
Normal	523 (53.4)	499 (55.0)	24 (33.8)	
Intense	186 (19.0)	142 (15.6)	44 (62.0)	
Job security				
Secure	363 (37.1)	347 (38.2)	16 (22.5)	**< 0.001**
Normal	440 (44.9)	409 (45.0)	31 (43.7)	
Insecure	176 (18.0)	152 (16.7)	24 (33.8)	
Working environment satisfaction				
Satisfied	386 (39.4)	376 (41.4)	10 (14.1)	**< 0.001**
Normal	513 (52.4)	468 (51.5)	45 (63.4)	
Dissatisfied	80 (8.2)	64 (7.0)	16 (22.5)	
Relationship with co-workers				
Good	238 (24.3)	225 (24.8)	13 (18.3)	**< 0.001**
Normal	525 (53.6)	499 (55.0)	26 (36.6)	
Not good	216 (22.1)	184 (20.3)	32 (45.1)	
Appreciation/reward system satisfaction				
Satisfied	246 (25.1)	241 (26.5)	5 (7.0)	**< 0.001**
Normal	476 (48.6)	442 (48.7)	34 (47.9)	
Dissatisfied	257 (26.3)	225 (24.8)	32 (45.1)	
Change in feeling overloaded before and during the COVID-19 outbreak				
Less overload	46 (4.7)	46 (5.1)	0 (0)	**< 0.001**
No change	522 (53.3)	500 (55.1)	22 (31.0)	
More overload	411 (42.0)	362 (39.9)	49 (69.0)	

*p-value from Pearson’s Chi-squared and Fisher’s exact test

About half of CHWs participated daily in tracing close contacts of confirmed COVID-19 cases (49.1%) and screening for COVID-19 symptoms in the community (53.7%) during the Tet holiday outbreak. More than one-third of CHWs daily organized isolation places for suspected cases (37.1%), while 32% worked on other activities with exposure to potential of SARS-CoV-2 sources. CHWs who did daily contact tracing or organized isolation places had higher rates of deteriorating to high depression levels compared to those who did not (62% vs. 48.1% and 46.5% vs. 36.3%, respectively). CHWs who worked in provinces with more recorded COVID-19 cases during the Tet holiday outbreak had higher percentages of deteriorating to high depression levels compared to those worked in provinces with less cases, though not on a statistically significant level (Table 3).

During the 2021 Tet holiday outbreak, respondents who deteriorated to high depression levels had higher percentages of poor sleep in both quantity and quality than non-deteriorating participants (sleeping less than seven hours per day: 87.3% vs. 48.8%; report quality of sleep as normal: 47.9% vs. 28.3%; report quality of sleep as not good: 38% vs. 10.1%). In general, working overtime and work on weekends were common among CHWs (87.6% and 88.8%, respectively). The percentages who worked in two or more places and who worked at least nine hours per day was nearly double among CHWs deteriorating to high depression levels compared to non-deteriorating CHWs (25.4% vs. 13.7% and 71.8% vs. 38.9%). While 15.6% of participants who did not deteriorate to high depression levels considered their jobs intense during the 2021 Tet holiday outbreak, this figure was 62% among the ones who did deteriorate to high depression levels. CHWs deteriorating to high depression levels were more likely to work in less favorable environments, i.e., in more insecure jobs (33.8% vs. 16.7%), were more dissatisfied with the working environment (22.5% vs. 7%), had worse relationship with their co-workers (45.1% vs. 20.3%), and were more dissatisfied with the appreciation/reward system (45.1% vs. 24.8%). They also felt more overloaded during the COVID-19 outbreak than non-deteriorating CHWs (69% vs. 39.9%) (Table 4).

In the univariate regression analysis (see Table 5), younger participants with fewer years of job experience and higher SES had higher odds of deterioration to high depression levels compared to older participants with more experience [age, OR (95% CI): 0.97 (0.94–0.99); years of job experience (ref: < 5 years), 5–14 years: 0.53 (0.29–0.96), 15 years and above: 0.44 (0.23–0.86)]; and lower SES [ref: upper and middle SES, lower SES: 2.02 (1.24–3.28)]. Both the Base Model and Model 1 confirmed the association between SES and deterioration to high depression levels, but showed weakened effects of age and years of job experience. Model 2 and Model 3 turned all above-mentioned associations statistically insignificant. CHWs with pre-existing long-term health problems and mental health disorders had increased odds to develop high depression levels in all of our models, though not at statistically significant levels. CHWs with higher exposure intensity during contact tracing had a 1.86-fold increase in the odds of deterioration to high depression levels (OR: 1.86, 95% CI: 1.02–3.41) and 1.81 times in the odds of deterioration when it came to organizing isolation (OR: 1.81, 95% CI: 1.03–3.2) in the unadjusted analysis compared with CHWs with a lower work-related SARS-CoV-2 exposure intensity. After adjusting for socio-demographic factors, pre-existing health conditions, and sleep/work conditions (Model 1 and Model 3), these associations were weakened to statistically insignificant levels. All other activities related to exposure intensity remained not associated on statistically significant levels in all our models.

**Table 5:** Factors related to deterioration to high depression levels among study participants

Intercepts	*n* (%)^a^	Univariate associations			Base model*			Model 1**			Model 2***			Model 3****		
		Odds ratio	95% CI	*p*-value	Odds ratio	95% CI	*p*-value^†^	Odds ratio	95% CI	*p*-value^†^	Odds ratio	95% CI	*p*-value^†^	Odds ratio	95% CI	*p*-value^†^
Sociodemographic																
Age [median, IQR]	35 (31, 42)	0.97	0.94–0.99	0.038	0.98	0.93–1.03	0.465	0.97	0.92–1.03	0.324	0.99	0.93–1.05	0.638	0.98	0.93–1.05	0.612
Sex							0.940			0.986			0.726			0.682
Male	35 (49.3)	Ref.			Ref.			Ref.			Ref.			Ref.		
Female	36 (50.7)	0.95	0.58–1.53	0.822	1.02	0.62–1.69		1.00	0.59–1.71		1.11	0.62–1.98		1.13	0.62–2.07	
Marital status							0.902			0.923			0.795			0.710
Single and divorced	10 (14.1)	Ref.			Ref.			Ref.			Ref.			Ref.		
Married	61 (85.9)	0.70	0.35–1.42	0.328	0.95	0.43–2.11		0.96	0.42–2.19		1.13	0.45–2.80		1.19	0.47–3.02	
Living with children or elderly							0.316			0.430			0.542			0.633
No	19 (26.8)	Ref.			Ref.			Ref.			Ref.			Ref.		
Yes	52 (73.2)	1.45	0.84–2.50	0.177	1.35	0.74–2.45		1.27	0.69–2.34		1.23	0.63–2.42		1.18	0.59–2.35	
Years of job experiences							0.273			0.231			0.314			0.270
< 5 y	19 (26.8)	Ref.			Ref.			Ref.			Ref.			Ref.		
5–14 y	32 (45.1)	0.53	0.29–0.96	0.035	0.57	0.29–1.12		0.54	0.27–1.08		0.56	0.25–1.23		0.51	0.23–1.16	
15 y +	20 (28.2)	0.44	0.23–0.86	0.015	0.63	0.22–1.82		0.60	0.20–1.77		0.42	0.12–1.49		0.41	0.11–1.54	
Social economic status							0.021			0.023			0.732			0.770
Upper and middle	36 (50.7)	Ref.			Ref.			Ref.			Ref.			Ref.		
Lower	35 (49.3)	2.02	1.24–3.28	0.005	1.82	1.10–3.02		1.82	1.09–3.05		1.11	0.6–2.05		1.10	0.59–2.04	
Health condition																
Long-term health problems							0.575			0.717			0.722			0.787
No	59 (83.1)	Ref.			Ref.			Ref.			Ref.			Ref.		
Yes	12 (16.9)	1.15	0.60–2.20	0.663	1.23	0.60–2.50		1.14	0.56–2.36		1.16	0.52–2.55		1.12	0.50–2.52	
Mental health disorders							–			–			–			–
No	65 (92.9)	Ref.			Ref.			Ref.			Ref.			Ref.		
Yes	5 (7.1)	2.42	0.90–6.47	0.079	2.11	0.77–5.78		2.26	0.8–6.39		1.65	0.52–5.26		1.85	0.56–6.13	
Intensity of direct exposure to SARS-CoV-2																
Contact tracing										0.346						0.826
Lower	15 (21.1)	Ref.						Ref.						Ref.		
Middle	12 (16.9)	1.14	0.52–2.49	0.739				0.91	0.37–2.22					0.75	0.26–2.11	
Higher	44 (62.0)	1.86	1.02–3.41	0.045				1.5	0.75–3.01					0.97	0.43–2.16	
Organizing isolation										0.478						0.805
Lower	21 (29.6)	Ref.						Ref.						Ref.		
Middle	17 (23.9)	1.57	0.81–3.04	0.185				1.45	0.69–3.07					0.83	0.35–1.97	
Higher	33 (46.5)	1.81	1.03–3.20	0.039				1.46	0.73–2.9					1.11	0.50–2.44	
Screening and testing										0.967						0.888
Lower	15 (21.1)	Ref.						Ref.						Ref.		
Middle	14 (19.7)	1.22	0.57–2.59	0.609				1.11	0.48–2.58					0.82	0.31–2.16	
Higher	42 (59.2)	1.39	0.75–2.55	0.291				1.08	0.52–2.25					0.83	0.36–1.92	
Other exposure activities										0.407						0.362
Lower	29 (40.8)	Ref.						Ref.						Ref.		
Middle	18 (25.4)	1.60	0.87–2.96	0.133				1.60	0.81–3.15					1.62	0.72–3.65	
Higher	24 (33.8)	1.28	0.73–2.24	0.392				1.16	0.63–2.14					0.89	0.43–1.84	
Distribution of confirmed cases in provinces										0.134						0.838
No case	47 (66.2)	Ref.						Ref.						Ref.		
Low number of cases	15 (21.1)	1.79	0.97–3.30	0.062				1.83	0.95–3.52					1.13	0.54–2.37	
High number of cases	9 (12.7)	1.51	0.72–3.20	0.276				1.68	0.75–3.76					0.82	0.33–2.04	
Sleep condition																
Hours of sleep per day													0.016			0.021
7 + hours	9 (12.7)	Ref.									Ref.			Ref.		
Less than 7 h	62 (87.3)	7.23	3.55–14.72	< 0.001							2.55	1.14–5.69		2.51	1.11–5.69	
Quality of sleep													0.044			0.042
Good	10 (14.1)	Ref.									Ref.			Ref.		
Normal	34 (47.9)	7.40	3.60–15.20	< 0.001							2.36	1.03–5.40		2.45	1.05–5.69	
Not good	27 (38.0)	16.41	7.69–35.02	< 0.001							3.09	1.21–7.92		3.17	1.21–8.29	
Work condition																
Working hours													0.879			0.890
< 8 h	6 (8.5)	Ref.									Ref.			Ref.		
8–9 h	14 (19.7)	1.70	0.64–4.49	0.284							1.02	0.33–3.12		1.06	0.34–3.32	
9 + hours	51 (71.8)	5.63	2.38–13.34	< 0.001							1.21	0.41–3.52		1.23	0.41–3.68	
Work overtime in workdays													0.426			0.476
No	4 (5.6)	Ref.									Ref.			Ref.		
Yes	67 (94.4)	2.48	0.89–6.92	0.083							0.53	0.12–2.40		0.57	0.12–2.60	
Work on weekends													0.293			0.288
No	1 (1.4)	Ref.									Ref.			Ref.		
Yes	70 (98.6)	9.55	1.31–69.44	0.026							3.50	0.28–42.88		3.53	0.29–43.18	
Working in two places +													0.050			0.044
No	53 (74.6)	Ref.									Ref.			Ref.		
Yes	18 (25.4)	2.15	1.22–3.79	0.008							1.92	1.01–3.74		2.05	1.04–4.03	
Work intensity													< 0.001			< 0.001
Not intense	3 (4.2)	Ref.									Ref.			Ref.		
Normal	24 (33.8)	4.28	1.28–14.35	0.018							1.36	0.36–5.19		1.36	0.35–5.29	
Intense	44 (62.0)	27.58	8.41–90.39	< 0.001							5.40	1.37–21.29		5.24	1.28–21.52	
Work security													0.707			0.704
Secure	16 (22.5)	Ref.									Ref.			Ref.		
Normal	31 (43.7)	1.64	0.88–3.06	0.116							0.73	0.35–1.53		0.73	0.34–1.54	
Insecure	24 (33.8)	3.42	1.77–6.63	< 0.001							0.78	0.33–1.86		0.78	0.32–1.87	
Working environment satisfaction													0.635			0.637
Satisfied	10 (14.1)	Ref.									Ref.			Ref.		
Normal	45 (63.4)	3.62	1.80–7.27	< 0.001							1.43	0.62–3.32		1.45	0.62–3.38	
Dissatisfied	16 (22.5)	9.40	4.09–21.63	< 0.001							1.65	0.54–5.04		1.65	0.53–5.12	
Relationship with co-workers													0.014			0.014
Good	13 (18.3)	Ref.									Ref.			Ref.		
Normal	26 (36.6)	0.90	0.45–1.79	0.767							1.23	0.56–2.72		1.15	0.51–2.58	
Not good	32 (45.1)	3.01	1.54–5.90	0.001							2.82	1.23–6.45		2.77	1.19–6.42	
Appreciation/reward system satisfaction													0.146			0.151
Satisfied	5 (7.0)	Ref.									Ref.			Ref.		
Normal	34 (47.9)	3.71	1.43–9.60	0.007							2.59	0.86–7.74		2.61	0.85–8.01	
Dissatisfied	32 (45.1)	6.86	2.63–17.90	< 0.001							2.87	0.91–9.04		2.91	0.91–9.37	
Feeling more overloaded during COVID-19													0.029			0.044
No	22 (31.0)	Ref.									Ref.			Ref.		
Yes	49 (69.0)	3.36	2.00–5.65	< 0.001							1.95	1.06–3.58		1.88	1.01–3.51	

*Base model: socio-demographic + pre-existing health condition

**Model 1: base model + direct exposure ***Model 2: base model + sleep/work condition ****Model 3: base model + direct exposure + sleep/work condition

^†^*p*-value from likelihood-ratio test

^a^Frequency and percentage of CHWs’ groups of deterioration to high depression levels

All our models showed that individuals who slept less than seven hours or who reported poor quality of sleep were more likely to deteriorate to high depression levels [Model 2: sleep less than seven hours: (ref: sleep 7 + hours) 2.55, 95% CI: 1.14–5.69; quality of sleep (ref: good), normal: 2.36, 95% CI: 1.03–5.40, not good: 3.09, 95% CI: 1.21–7.92]. Compared with CHWs who worked less than eight hours per day, those who worked more than nine hours per day had a 5.6-fold increase in the odds of deteriorating to high depression levels in univariate analysis, however these odds reduced to 1.2 after adjusting for socio-demographic factors, pre-existing health condition, and SARS-CoV-2 exposure intensity (Model 2 and Model 3).

In univariate analysis, experiencing poor working conditions such as work on weekends, work at more than one workplace, intense and insecure work, dissatisfaction with the work environment and the appreciation/reward system, poor relationship with co-workers, and feeling more overloaded than before COVID-19 were associated with higher odds of deterioration on statistically significant levels compared with those who did not. These above-mentioned relationships were confirmed in multivariable analysis, however only CHWs who worked at more than one workplace with high intensity, poor relationship with co-workers, and feeling more overloaded than before COVID-19 remained with increased odds of deterioration to high depression levels at a statistically significant level (Model 2 and Model 3).

Both AIC and BIC indicated that Model 2, which included socio-demographic factors, pre-existing health conditions, and sleep/work conditions, was the best model fit (AIC = 413.8, BIC = 555.5) (Table 6).

**Table 6:** Comparison of logistic regression models

Model	Matrix				
	Log likelihood of null model	Log likelihood of full model	Degrees of freedom (df)	AIC^†^	BIC^†^
Base model*	− 252.02	− 243.39	10	506.78	555.64
Model 1**	− 252.02	− 236.27	20	512.55	610.26
Model 2***	− 252.02	− 177.91	29	413.83	555.51
Model 3****	− 252.02	− 176.30	39	430.61	621.14

^†^AIC: Akaike’s Information Criteria; BIC: Schwarz’s Bayesian Information Criteria

*Base model: socio-demographic + pre-existing health condition

**Model 1: base model + direct exposure ***Model 2: base model + sleep/work condition ****Model 3: base model + direct exposure + sleep/ work condition

## Discussion

To our knowledge, this was the first study worldwide to quantify the effects of COVID-19-related work on depression levels among CHWs. We found a substantial increase in depression across all severity levels among CHWs during the 2021 Tet holiday outbreak compare to pre-pandemic baseline levels that was likely attributable to their COVID-19-related work. Our findings also indicated that CHWs who reported poor sleep conditions and worked in unfavorable working environments might be at particular risk of deterioration to high depression levels. CHWs involved in contact tracing and the organization of quarantine for suspect cases were also at increased risk of deterioration, however, these associations weakened after adjusting for socio-demographic factors, pre-existing health conditions, and sleep/work conditions.

Our results of the effects of COVID-19-related work among CHWs, a cadre of the health workforce that is involved in non-clinical public health activities at community level in many low- and middle-income countries, are comparable with findings from studies among frontline clinical staff caring for COVID-19 patients. A large study among health care workers in China using the Hospital Anxiety and Depression Scale reported 36.1% of medical staff as suffering from depression [22]. Another study from China that used the same PHQ-9 questionnaire as in our study estimated the depression levels among clinical staff treating COVID-19 patients at 57.6% [26]. A pooled analysis of 57 cross-sectional studies using self-reported standardized questionnaires to assess depressive symptoms estimated a prevalence of depression of 43% (range 14.3–99.5%) among health care workers with direct contact to COVID-19 patients [7]. The fact that the prevalence of depression among CHWs in our study was even higher than this summary estimate points to the unrecognized burden of CHWs and other non-clinical public health staff during the COVID-19 pandemic.

The psychological response of heath care workers to the pandemic is complex [27]. We found CHWs in Vietnam to be at substantial risk of infection because of their close, frequent contact with potential SARS-CoV-2 sources as well as working longer hours than usual and even working on the weekends. Most CHWs in our study who deteriorated to high depression levels were relatively young, had few years of work experience, low SES, and poor sleep conditions that also contributed to the deterioration. Health workers in Vietnam do usually not receive mental health training, which might have contributed to this development. Particular attention is warranted regarding the mental health well-being of CHWs, especially those working in areas with substantial community transmission. In our study, CHWs with high frequencies of exposure to potential SARS-CoV-2 cases were more likely to deteriorate to high depression levels. This is congruent with evidence from Ethiopia and China among clinical health care workers [5, 28].

The importance of a healthy workplace to support mental health is evident from the scientific literature [29, 30] as well as our results. Our findings highlight that certain factors that might increase the risk for deterioration to high depression levels during the COVID-19 pandemic include work intensity, insecure work conditions, dissatisfied working environments and reward systems, and poor relationships with co-workers. These risks are unlikely to act in an isolated manner, but, instead, typically interact to exponentially elevate the risks of depression [31]. Such evidence is needed to inform health policy to provide more targeted psychological care for CHWs in the COVID-19 pandemic in Vietnam and elsewhere, as well as to inform health workforce planning for improved community-based response to other infectious disease outbreaks in the future.

Noteworthy strengths of our study include the use of a validated questionnaire, a relatively large sample size, and that we were able to compare depression at two time points in the same population, before the start of the COVID-19 pandemic and during the height of a nationwide outbreak about one year into the pandemic, while most other studies only asked about depression symptoms at one single time point without comparisons. Furthermore, our target population was novel—CHWs, an important cadre of frontline workers in most low- and middle-income countries who perform crucial tasks during the response to COVID-19, but who are often overlooked in occupational mental health interventions. Indeed, all current evidence investigating work-related depression was done among clinical health care workers working in health facilities.

We acknowledge certain limitations of our study. First, reducing our originally 1–30 scaled continuous outcome variable into five and two categories resulted inevitably in a loss of variability. We did so in order to identify those CHWs who might qualify for priority mental health interventions. Second, information on depression symptoms and all covariates were self-reported. Third, recall bias for the pre-COVID period might have affected our results. Since recall is known to improve when following an ordered sequence of events [32], we structured our outcome assessment by starting with questions about the pre-pandemic period first before asking about the Tet holiday period, thereby attempting to reduce recall bias (see Additional file 1). However, given the considerable length of the recall period, we acknowledge the potentially high likelihood of recall bias for the questions relating to the pre-pandemic period. Unfortunately, no evidence exists on pre-pandemic depression levels using the PHQ-9 questionnaire among CHWs working in public health in Vietnam and in other settings worldwide that could serve as reference. Fourth, sleep and work condition were only captured for the period of the 2021 Tet holiday outbreak, hence we could not rule out reverse causality effects between certain covariates like unhealthy working conditions and poor sleep conditions with deterioration to high depression levels. Fifth, as participation was voluntary and the sample was self-selected, there might have been differences among CHWs who decided to participate compared to those who either did not. We were not able to assess the extent of this potential selection bias. Sixth, because we designed the questionnaire to be completed within 15 min, we might have missed to collect other potentially relevant factors such as feelings of vulnerability or loss of control, concerns about health, the spread of the virus, or the health of family. Further qualitative research might be needed to provide complementary evidence.

## Conclusions

We found a substantial increase in depression levels among CHWs in Vietnam due to their COVID-19 related work. In particular, the 4- to 5-fold increase in CHW suffering from severe depression levels is worrisome. CHWs are an indispensable yet often overlooked cadre of work in many low- and middle-income countries and shoulder a heavy psychological burden during the COVID-19 pandemic due to the nature of their work. Targeted psychological support for CHWs is crucial to improve mental health and ensure sustainability of community-based health interventions during COVID-19 and future epidemics.

## Supplementary Information

**Additional file 1. Supplement 1**. Questionnaire in english version.

**Additional file 2. Supplement 2**. Details of variable management in this project.

## Data Availability

The datasets used and/or analyzed during the current study are available from the corresponding author (NAH) on reasonable request.

## Abbreviations

CHWs: Community health workers
PHQ-9: Patient Health Questionnaire
SES: Socio-economic status
95% CIs: 95% Confidence intervals
ORs: Odds ratios
AIC: Akaike’s information criteria
BIC: Schwarz’s Bayesian information criteria
NIHE: National Institute of Hygiene and Epidemiology
IQR: Interquartile range
