# Supplementary material for: Assessing the mental effects of COVID-19-related work on depression among community health workers in Vietnam
Source: Hum Resour Health. 2022 Aug 19;20:64. doi: 10.1186/s12960-022-00760-x (PMC9390118; doi:10.1186/s12960-022-00760-x)
Supplement: Supplementary file 1 — Additional file 1. Supplement 1. Questionnaire in english version. [file 12960_2022_760_MOESM1_ESM.docx]

**Supplement 1:**

**1.1. QUESTIONNAIRE IN ENGLISH VERSION**

**A. INCLUSION/EXCLUSION CRITERIA**

| A1. Did you participate in contact tracing/case finding for COVID-19 confirmed cases or suspected cases in the 2021 Tet holiday outbreak? | 1. Yes  2. No |
| --- | --- |
| *A1.1. If yes,*  In general, how often did you perform these activities? | 1. Daily  2. > 3 days per week  3. 1-2 days per week  4. Less than once per week |
| A2. Did you participate in organizing isolation for COVID-19 confirmed cases and/or organizing quarantine for COVID-19 suspected cases in the 2021 Tet holiday outbreak? | 1. Yes  2. No |
| *A2.1. If yes,*  In general, how often you did perform these activities? | 1. Daily  2. > 3 days per week  3. 1-2 days per week  4. Less than once per week |
| A3. Did you participate in screening for COVID-19 symptoms in the community in the 2021 Tet holiday outbreak?  *(for example: taking SARS-CoV-2 swab samples, interviewing people for clinical symptoms, or measuring temperature)* | 1. Yes  2. No |
| *A3.1. If yes,*  In general, how often you did these activities? | 1. Daily  2. > 3 days per week  3. 1-2 days per week  4. Less than once per week |
| A4. **If the answer is No for all three above questions,** did you participate in any other activity which involved direct exposure to potential SARS-CoV-2 sources in the 2021 Tet holiday outbreak?  *(If yes, specify)* | 1. Yes  2. No |
| *A4.1. If yes,*  In general, how often you did these activities? | 1. daily  2. > 3 days per week  3. 1-2 days per week  4. less than once per week |

**B. GENERAL INFORMATION**

| **B1. DEMOGRAPHICS** |  |
| --- | --- |
| B1.1. During the 2021 Tet holiday outbreak, what was the level in the health system you were working in? | 1. National level (move B1.3)  2. Provincial level  3. District level  4. Commune and lower levels |
| B1.2. During 2021 Tet holiday outbreak, what was the province/city you were working in?  *(Skip this question for CHWs at national level)* | Dropdown a list of 64 provinces and cities in Vietnam |
| B1.3. Age |  |
| B1.4. Sex | 1. Male  2. Female |
| B1.5. Marital status | 1. Single  2. Married  3. Others |
| B1.6. Living with children under 5 or elders (65+) in the household | 1. Yes  2. No |
| B1.7. Years of job experience in preventive medicines and public health | (years) |
| B1.8. Subjective social economic status | 1. High  2. Middle  3. Low-middle  4. Low |
| **B2. HEALTH CONDITION** |  |
| B2.1. Do you have any long-term health problems, like heart disease, diabetes etc | 1. Yes  2. No **(Move to B.2.2)** |
| B2.1.1. Which long-term health problem do you have?  (Multiple choices) | 1. COPD  2. Asthma  3. Rheumatoid arthritis  4. High blood pressure  5. Mental health disorders (Depression/Stress/Anxiety) (skip question B2.2)  6. Hepatitis  7. Obesity  8. Diabetes  9. Chronic kidney disease  10. Any types of cancers  11. Others (specify) |
| B2.2. Do you have any mental health disorders which has been diagnosed by certified doctors before?  *(If yes, specify)* | 1. Yes  2. No **(Move to B2.3)** |
| B2.2.1. Which pre-existing mental health disorders do you have?  (Multiple choices) | 1. Depression  2. Bipolar disorders  3. Schizophrenia  4. Amnesia  5. Personality disorders  6. Others (specify) |
| B2.3. Have you had any acute/sudden-onset medical problems since the beginning of the 2021 Tet outbreak?  *(If yes, specify)* | 1. Yes  2. No |
| B.2.3.1. Which acute/sudden-onset medical problems do you have?  (Multiple choices) | 1. Stroke  2. Heart attack  3. Brain injury  4. Bone fractures  5. Any types of burns  6. Others (specify) |
| **B3. SLEEP CONDITION** |  |
| B3.1. Do you have enough time to sleep (at least 7 hours) in the 2021 Tet holiday outbreak? | 1. Yes  2. No |
| B3.2. Please describe the rest you obtain during your night sleep | 1. Good  2. Normal  3. Not good  4. Bad |

**C. WORKING CONDITIONS**

| C1. In general, how many hours did you usually work per day in the 2021 Tet holiday outbreak (from February 2021)? | 1. <7 hours  2. 7-8 hours  3. 9-10 hours  4. >10 hours |
| --- | --- |
| C2. Did you work overtime in the 2021 Tet holiday outbreak (from February 2021)?  *(bonus at least 2 hours in either shift work or night work in at least two days in a week)* | 1. Yes  2. No |
| C3. Did you work at weekends in the 2021 Tet holiday outbreak (from February 2021)?  *(bonus at least 4 days in a month)* | 1. Yes  2. No |
| C4. In general, how intense was your job in the 2021 Tet holiday outbreak? | 1. Very intense, I cannot complete 50% of tasks during normal work time  2. Intense, I cannot complete 75% of tasks during normal work time  3. OK, I can control all tasks during work time  4. Not intense, I feel I could do many more tasks |
| C5. Do you have to work in more than one workplace in the 2021 Tet holiday outbreak?  *Note: Only consider where pay wages for you* | 1. Yes  2. No |
| C6. How secure do you feel in your job in the 2021 Tet holiday outbreak? | 1. Highly secure  2. Secure  3. OK  4. Insecure |
| C7. In general, how do you feel about your working environment in the 2021 Tet holiday outbreak? | 1. Highly satisfied  2. Satisfied  3. OK  4. Dissatisfied |
| C8. In general, what is the quality of the work relationship with your employers and co-workers in the 2021 Tet holiday outbreak? | 1. Very good  2. Good  3. OK  4. Bad |
| C9. Did you receive any COVID-19 related monetary welfare? | 1. Yes  2. No |
| C10. Are you satisfied with the appreciation or reward system in the 2021 Tet holiday outbreak? | 1. Highly satisfied  2. Satisfied  3. OK  4. Dissatisfied |

**D. WORKLOAD**

| D1. On average, how often did you feel overloaded in the 2021 Tet holiday outbreak? | 1. Often  2. Sometimes  3. Almost never  4. Never |
| --- | --- |
| D2. On average, how often did you feel overloaded in six months before COVID-19 pandemic (from June 2019 to December 2019)? | 1. Often  2. Sometimes  3. Almost never  4. Never |

**F. DEPRESSION SYMPTOMS AS PER PATIENT HEALTH QUESTIONNAIRE 9**

How often have you experienced any of the following problems?

|  | **On average during the six months before COVID-19 pandemic** | | | | **2021 Tet holiday outbreak** | | | |
| --- | --- | --- | --- | --- | --- | --- | --- | --- |
|  | >4 days/week | 3-4 days/week | 1-2 days/week | Not at all | >4 days/week | 3-4 days/week | 1-2 days/week | Not at all |
| F1. Little interest or pleasure in doing things | 0 | 1 | 2 | 3 | 0 | 1 | 2 | 3 |
| F2. Feeling down, depressed, or hopeless | 0 | 1 | 2 | 3 | 0 | 1 | 2 | 3 |
| F3. Trouble falling or staying asleep, or sleeping too much | 0 | 1 | 2 | 3 | 0 | 1 | 2 | 3 |
| F4. Feeling tired or having little energy | 0 | 1 | 2 | 3 | 0 | 1 | 2 | 3 |
| F5. Poor appetite or overeating | 0 | 1 | 2 | 3 | 0 | 1 | 2 | 3 |
| F6. Feeling bad about yourself or that you are a failure or have let yourself or your family down | 0 | 1 | 2 | 3 | 0 | 1 | 2 | 3 |
| F7. Trouble concentrating on things, such as reading the newspaper or watching television | 0 | 1 | 2 | 3 | 0 | 1 | 2 | 3 |
| F8. Moving or speaking so slowly that other people could have noticed. Or the opposite being so fidgety or restless that you have been moving around a lot more than usual | 0 | 1 | 2 | 3 | 0 | 1 | 2 | 3 |
| F9. Thoughts that you would be better off dead, or of hurting yourself | 0 | 1 | 2 | 3 | 0 | 1 | 2 | 3 |

**1.2. QUESTIONNAIRE IN VIETNAMESE VERSION**

**A. Thông tin chung:**

| A1. Anh/Chị có tham gia điều tra truy vết các trường hợp tiếp xúc với ca dương tính COVID-19 tại cộng đồng trong đợt dịch COVID-19 Tết Tân Sửu không? | 1. Có  2. Không |
| --- | --- |
| ***A1.1. Anh/Chị có thường xuyên thực hiện công việc đó không?*** | 1. Hàng ngày  2. >3 ngày/tuần  3. 1-2 ngày/tuần  4. Ít hơn 1 ngày/tuần |
| A2. Anh/Chị có thưc hiện tổ chức cách ly ca nghi ngờ hoặc ca khẳng định mắc COVID-19 trong đợt dịch COVID-19 Tết Tân Sửu không?  *(Bao gồm cả hoạt động cách ly những người nước ngoài nhập cảnh vào Việt Nam)* | 1. Có  2. Không |
| ***A2.1. Anh/Chị có thường xuyên thực hiện công việc đó không?*** | 1. Hàng ngày  2. >3 ngày/tuần  3. 1-2 ngày/tuần  4. Ít hơn 1 lần/tuần |
| A3. Anh/Chị có tham gia sàng lọc COVID-19 tại cộng đồng trong đợt dịch COVID-19 Tết Tân Sửu không?  *(ví dụ: lấy mẫu các đối tượng nghi ngờ và đo nhiệt độ tại các điểm công cộng)* | 1. Có  2. Không |
| ***A2.3. Anh/Chị có thường xuyên thực hiện công việc đó không?*** | 1. Hàng ngày  2. >3 ngày/tuần  3. 1-2 ngày/tuần  4. Ít hơn 1 lần/tuần |
| A4. Anh/Chị có tham gia vào bất kỳ hoạt động nào khác có nguy cơ tiếp xúc với nguồn lây nhiễm COVID-19 trong đợt dịch COVID-19 Tết Tân Sửu không? | 1. Có  2. Không |
| ***A4.1. Vui lòng liệt kê tên các hoạt động đó*** |  |
| ***A4.2. Anh/Chị có thường xuyên thực hiện công việc đó không?*** | 1. Hàng ngày  2. >3 ngày/tuần  3. 1-2 ngày/tuần  4. Ít hơn 1 lần/tuần |

**B. THÔNG TIN CỦA NGƯỜI THAM GIA**

| **B1. THÔNG TIN NHÂN KHẨU HỌC** |  |
| --- | --- |
| B1.1. Hiện nay, Anh/Chị đang công tác tại tuyến nào?  *(Vui lòng chọn tuyến cao nhất)* | 1. Tuyến trung ương (Chuyển B1.3)  2. Tuyến tỉnh  3. Tuyến huyện  4. Tuyến xã/dưới xã (thôn, bản, ấp…) |
| B1.2. Anh/chị đang công tác tại tỉnh/thành phố nào? | Danh sách 64 tỉnh thành |
| B1.3. Năm sinh dương lịch của Anh/Chị |  |
| B1.4. Giới tính của Anh/Chị | 1. Nam  2. Nữ |
| B1.5. Tình trạng hôn nhân của Anh/Chị | 1. Độc thân  2. Đã kết hôn  3. Khác (ly thân/ly hôn…) |
| B1.6. Anh/Chị có đang sống cùng nhà với người già (trên 65 tuổi) hoặc trẻ em dưới 5 tuổi không? | 1. Có  2. Không |
| B1.7. Số năm Anh/Chị công tác trong lĩnh vực Y tế công cộng | (năm) |
| B1.8. Anh/Chị thấy kinh tế gia đình mình đang ở mức nào? | 1. Khá  2. Trung bình  3. Trung bình - thấp  4. Thấp |
| **B2. TÌNH TRẠNG SỨC KHỎE** |  |
| B2.1. Hiện Anh/Chị có đang mắc bệnh lý mạn tính nào không? | 1. Có  2. Không |
| B2.1.1. Anh/Chị đang mắc bệnh lý mạn tính nào?  (Câu hỏi nhiều lựa chọn) | 1. Viêm phổi tắc nghẽn mạn tính (COPD)  2. Hen suyễn  3. Viêm khớp mạn  4. Cao huyết áp  5. Trầm cảm  6. Viêm gan mạn  7. Béo phì  8. Tiểu đường  9. Bệnh thận mạn  10. Ung thư  11. Khác (ghi rõ) |
| B2.1.1.1. Vui lòng liệt kê tên các bệnh lý mạn tính khác |  |
| B2.2. Anh/Chị đã từng mắc bất kì bệnh/triệu chứng sức khỏe tâm thần nào được chẩn đoán bởi nhân viên y tế chưa? | 1. Đã từng  2. Chưa bao giờ |
| B2.2.1. Anh/Chị đã từng mắc bệnh/triệu chứng sức khỏe tâm thần nào?  (Câu hỏi nhiều lựa chọn) | 1. Trầm cảm  2. Rối loạn cảm xúc lưỡng cực  3. Tâm thần phân liệt  4. Giảm/mất trí nhớ  5. Rối loạn phát triển  6. Khác (ghi rõ) |
| B2.2.1.1. Vui lòng liệt kê tên các bệnh/triệu chứng sức khỏe tâm thần khác |  |
| B2.3. Anh/Chị có bị bất kỳ tổn thương/chấn thương trên cơ thể nào tính từ khi bắt đầu đợt dịch COVID-19 Tết Tân Sửu (27/01/2021) không?  *ví dụ, gãy tay, chân* | 1. Có  2. Không |
| B2.3.1. Anh/Chị đã bị tổn thương/chấn thương nào?  (Câu hỏi nhiều lựa chọn) | 1. Đột quỵ  2. Nhồi máu cơ tim  3. Chấn thương sọ não  4. Chấn thương xương, mô mềm (ví dụ, gãy tay, chân)  5. Bỏng  6. Khác (ghi rõ) |
| B2.3.1.1. Vui lòng liệt kê các tổn thương/chấn thương khác |  |
| **B3. TÌNH TRẠNG GIẤC NGỦ** |  |
| B3.1. Trong đợt dịch COVID-19 Tết Tân Sửu, Anh/Chị có được ngủ có được ngủ tối thiểu 7 giờ/ngày không?  *hoặc ngủ ít hơn 7 giờ/ngày nhưng luôn ở trạng thái tỉnh táo mà không cần sự hỗ trợ của chất kích thích như cafe, trà...* | 1. Có  2. Không |
| B3.2. Chất lượng giấc ngủ của Anh/Chị trong đợt dịch COVID-19 Tết Tân Sửu? | 1. Tốt  2. Bình thường  3. Kém  4. Rất kém |

**C. ĐIỀU KIỆN LÀM VIỆC**

| C1. Thời gian làm việc trung bình 1 ngày trong đợt dịch COVID-19 Tết Tân Sửu?  *Tổng thời gian làm việc không chỉ riêng cho hoạt động phòng chống dịch COVID-19* | 1. <7 giờ  2. 7 - 8 giờ  3. 9 – 10 giờ  4. > 10 giờ |
| --- | --- |
| C2. Anh/Chị có phải làm việc ngoài giờ hành chính trong đợt dịch COVID-19 Tết Tân Sửu không?  *chọn Có nếu Anh/Chị làm thêm ít nhất 2 giờ/ngày và ít nhất 2 ngày/tuần)* | 1. Có  2. Không |
| C3. Anh/Chị có phải làm việc vào Thứ 7 và/hoặc Chủ nhật trong đợt dịch COVID-19 Tết Tân Sửu không?  *chọn Có nếu Anh/Chị làm ít nhất 4 ngày Thứ 7/Chủ nhật trong tháng* | 1. Có  2. Không |
| C4. Cường độ công việc của Anh/Chị trong đợt dịch COVID-19 Tết Tân Sửu như thế nào?  *Chọn "Rất căng thẳng" nếu không thể hoàn thành được 1 nửa khối lượng công việc cần làm trong 8 tiếng; "Không căng thẳng" nếu kiểm soát công việc tốt* | 1. Rất căng thẳng  2. Căng thẳng  3. Bình thường  4. Không căng thẳng |
| C5. Anh/Chị có làm việc kiêm nhiệm ở cơ quan nào khác trong đợt dịch COVID-19 tết Tân Sửu không?  *(chỉ tính các đơn vị mà Anh/Chị được trả lương khi làm việc)* | 1. Có  2. Không |
| C6. Anh/Chị có cảm thấy an toàn khi làm việc trong đợt dịch COVID-19 tết Tân Sửu không? | 1. Rất an toàn  2. An toàn  3. Bình thường  4. Không an toàn |
| C7. Anh/Chị có hài lòng với môi trường làm việc trong đợt dịch COVID-19 tết Tân Sửu không? | 1. Rất hài lòng  2. Hài lòng  3. Bình thường  4. Không hài lòng |
| C8. Anh/Chị cảm thấy mối quan hệ với các đồng nghiệp trong đợt dịch COVID-19 tết Tân Sửu như thế nào? | 1. Rất tốt  2. Tốt  3. Bình thường  4. Không tốt |
| C9. Anh/Chị có được hỗ trợ thêm kinh phí liên quan tới các hoạt động phòng chống dịch COVID-19 không? | 1. Có  2. Không |
| C10. Anh/Chị có hài lòng với chế độ đãi ngộ (cơ chế thưởng/phạt) trong hoạt động phòng chống dịch COVID-19 không? | 1. Rất hài lòng  2. Hài lòng  3. Bình thường  4. Không hài lòng |

**D. KHỐI LƯỢNG CÔNG VIỆC**

| D1. Anh/Chị có thường xuyên cảm thấy quá tải bởi khối lượng công việc trong đợt dịch COVID-19 tết Tân Sửu (bắt đầu từ 27/01/2021) không? | 1. Thường xuyên  2. Thỉnh thoảng  3. Hiếm khi  4. Không bao giờ |
| --- | --- |
| D2. Trong vòng 6 tháng trước dịch COVID-19 (6/2019 – 12/2019), Anh/Chị có thường xuyên cảm thấy quá tải bởi khối lượng công việc không? | 1. Thường xuyên  2. Thỉnh thoảng  3. Hiếm khi  4. Không bao giờ |

**E. TÌNH TRẠNG TRẦM CẢM**

Vui lòng chọn tần suất Anh/Chị cảm thấy:

|  | **Trước dịch COVID-19** | | | | **Trong dịch COVID-19** | | | |
| --- | --- | --- | --- | --- | --- | --- | --- | --- |
|  | >4 ngày/tuần | 3-4 ngày/tuần | 1-2 ngày/tuần | Hoàn toàn không | >4 ngày/tuần | 3-4 ngày/tuần | 1-2 ngày/tuần | Hoàn toàn không |
| F1. Không hứng thú với các công việc thường ngày | 0 | 1 | 2 | 3 | 0 | 1 | 2 | 3 |
| F2. Buồn chán, dễ cáu giận | 0 | 1 | 2 | 3 | 0 | 1 | 2 | 3 |
| F3. Khó ngủ hoặc ngủ quá nhiều | 0 | 1 | 2 | 3 | 0 | 1 | 2 | 3 |
| F4. Cơ thể mệt mỏi/uể oải | 0 | 1 | 2 | 3 | 0 | 1 | 2 | 3 |
| F5. Chán ăn hoặc ăn quá nhiều | 0 | 1 | 2 | 3 | 0 | 1 | 2 | 3 |
| F6. Bản thân không làm được gì có ích | 0 | 1 | 2 | 3 | 0 | 1 | 2 | 3 |
| F7. Khó tập trung vào các hoạt động thông thường như đọc báo, xem tivi | 0 | 1 | 2 | 3 | 0 | 1 | 2 | 3 |
| F8. Cơ thể chậm chạm, ì ạch hoặc kích thích, vận động quá mức | 0 | 1 | 2 | 3 | 0 | 1 | 2 | 3 |
| F9. Có ý nghĩ đến tự tử hoặc tự làm tổn thương bản thân mà trước đây chưa từng có | 0 | 1 | 2 | 3 | 0 | 1 | 2 | 3 |
