# Supplementary material for: Assessing the mental effects of COVID-19-related work on depression among community health workers in Vietnam
Source: Hum Resour Health. 2022 Aug 19;20:64. doi: 10.1186/s12960-022-00760-x (PMC9390118; doi:10.1186/s12960-022-00760-x)
Supplement: Supplementary file 2 — Additional file 2. Supplement 2. Details of variable management in this project. [file 12960_2022_760_MOESM2_ESM.docx]

**Supplement 2: Details of variable management in this project**

*Depression levels*

We used the Vietnamese version of PHQ-9 to quantify depression levels among respondents. The PHQ-9 has been in widespread use around the world since its development in 1999 (12), and its Vietnamese version has been used in several previous studies(13–16). The PHQ-9 contains nine items about the frequency of depression-related symptoms. Each item has four response categories: “Not at all” (=0), “Several days” (=1), “More than half of the days” (=2), and “Nearly every day” (=3), resulting in a total range score from 0 to 27. The recommended cut-off points for depression thresholds are as follows: normal/minimal (0–4), mild (5–9), moderate (10–14), moderately severe (15–19), and severe (20–27) (17).

We asked self-reported depression levels at two time points: six months before the pandemic and during the 2021 Tet holiday outbreak. For regression models, the outcome variable "deterioration to high depression levels" was created. People who reported normal/minimal, mild, or moderate depression levels before the pandemic and an increase to moderately severe or severe levels during the 2021 Tet holiday outbreak; and those with moderately severe depression levels before the start of the pandemic and an increase to severe levels during the 2021 Tet holiday outbreak were categorized as having deteriorated to high depression levels.

*Socio-demographic characteristics*

Age was collected as a discrete variable and subsequently categorized into six groups: 18 to 29 years, 30 to 34 years, 35 to 39 years, 40 to 44 years, 45 to 49 years, or 50 years or older. Sex was defined as a binary variable for males and females. Marital status was comprised of three groups: single, married, or divorced/widowed. Living with children under the age of five or with elderly persons was defined as a binary variable (yes/no). Self-reported SES was captured in three levels: upper, middle, or lower. Years of experience in public health and preventive medicine was collected as a discrete variable and then categorized into six levels: less than 5 years, 5 to 9 years, 10 to 14 years, 15 to 19 years, 20 to 24 years, or 25 years or higher.

*Pre-existing health conditions*

We asked if respondents had been diagnosed with any pre-existing physiological or mental health conditions, or an acute medical issue (yes/no).

*Intensity of exposure to SARS-CoV-2 sources*

Work-related intensity of exposure to SARS-CoV2 was assessed through four questions on the estimated weekly frequencies of four activities: 1) the participation in contact tracing/case finding; 2) the organization of quarantine for suspected cases and close contacts; 3) the screening for COVID-19 symptoms in the community by taking swab samples or measuring temperature; and 4) any other activities that required direct exposure to potential SARS-CoV-2 cases. Answers were categorized into five levels for each question: not performed, performed less than one day per week, 1 to 2 days, 3 to 6 days, or daily. For regression models, exposure intensity was re-categorized into three groups: low (those who did not perform a particular activity), middle (performed less than one day, 1 to 2 days, or 3 to 6 days per week), and high (performed daily).

*Sleep condition*

We asked two questions about sleep conditions, including the estimated number of hours to sleep daily and the self-reported quality of sleep (good/normal/not good).

*Work condition*

Work condition referred to changes in feeling overloaded before the pandemic and during the 2021 Tet holiday outbreak (less, no change, more); estimated working hours per day; working overtime (defined as working at least two hours more than normal or work during night time unless stipulated), working at weekends (yes/no); and the number of paid workplaces for respondents. We also asked participants five questions on their overall perceived working environment: job intensity (not intense/normal/intense); job security (secure/normal/insecure); relationship with employers and co-works (good/normal/not good); satisfaction with the working environment; and the appreciation or reward system (satisfied/normal/dissatisfied).

*Case load during the 2021 Tet holiday outbreak*

Using the national COVID-19 incidence database, we used the number of confirmed COVID-19 cases per province during the 2021 Tet holiday outbreak to categorize CHWs into three groups: CHWs working in provinces with zero confirmed case; with low case numbers (<27 cases); or high case numbers (≥27 cases).
